# A Permutation Test for a Modified Spearman’s Rank Correlation Using Martingale-Residual Ranks Under Right Censoring

**DOI:** 10.21203/rs.3.rs-10168027/v1

**Published:** 2026-08-07

**Authors:** Alan D. Hutson, Han Yu

**Affiliations:** 1 Roswell Park Comprehensive Cancer Center, Department of Biostatistics and Bioinformatics, Elm and Carlton Streets, Buffalo, NY 14623

**Keywords:** permutation tests, Spearman correlation, martingale residuals, right-censored data, survival analysis

## Abstract

This note presents a robust permutation testing approach for assessing a modification of the Spearman rank correlation coefficient between right-censored variables. The method is based on univariate martingale residuals (UMRs), which provide a principled rank-generating transformation that incorporates censoring through the Kaplan–Meier estimators while reducing to the identity transformation when no censoring is present. Under the null hypothesis *H*_0_ : *ρ_s_* = 0, we apply the studentized permutation framework to the paired UMRs, yielding a test whose permutation distribution consistently estimates the sampling distribution of the statistic. The resulting procedure achieves asymptotically correct Type I error control, applies when one or both margins are censored, and reduces exactly to the classical Spearman permutation test in the absence of censoring. Simulation studies demonstrate strong Type I error control and competitive power across a range of censoring scenarios, and two real-data examples illustrate the method’s practical utility. Readily accessible R software implementations support the proposed test.

## 1 Introduction

Rank-based measures of association play a central role in nonparametric statistics, with Spearman’s rank correlation coefficient *ρ_s_*[1] being one of the most widely used. In the absence of censoring, *ρ_s_* is defined as the Pearson correlation between the marginal distribution functions *F_X_*(*X*) and *F_Y_*(*Y*), or equivalently, between the normalized ranks of the paired observations (*X_i_*, *Y_i_*), *i* = 1, …, *n*. Its robustness to outliers and its ability to capture general monotone association have made Spearman’s *ρ_s_* a standard tool across scientific disciplines.

When one or both variables are subject to right censoring, however, the construction of a rank-based measure of association becomes substantially more challenging. Several recent proposals[2] develop estimators of a “Spearman-like” correlation that reduce to the classical Spearman coefficient when censoring is absent. These approaches provide useful descriptive measures but do not yield a valid hypothesis test for *H*_0_ : *ρ_s_* = 0, nor do they guarantee correct Type I error control under censoring. Likewise, Kendall-type concordance measures adapted to censoring[3] provide a test of independence but do not target *ρ_s_* and are known to be conservative in many settings.

To the best of our knowledge, no existing method provides a hypothesis test for *H*_0_ : *ρ_s_* = 0 that (i) achieves asymptotically correct Type I error control under right censoring, (ii) applies when one or both margins are censored, and (iii) reduces exactly to the classical Spearman permutation test when censoring is absent. This gap is particularly important in biomedical applications, where paired survival outcomes are common and censoring is often substantial.

In this note, we introduce a simple and robust permutation test for *H*_0_ : *ρ_s_* = 0 based on the univariate martingale residuals (UMRs)[4]. The UMRs provide a natural rank-generating transformation for censored data, preserving the marginal information contained in the Kaplan–Meier estimators while reducing to the identity transformation when no censoring is present. By applying the studentized permutation framework[5] to the paired UMRs, we obtain a test statistic whose permutation distribution consistently estimates its sampling distribution under *H*_0_. This yields an asymptotically valid test with excellent finite-sample performance.

The remainder of the paper is organized as follows. Section 2 reviews the construction of paired UMRs. Section 3 defines the UMR-based Spearman correlation and presents the studentized permutation test. Section 4 reports simulation results demonstrating strong Type I error control and competitive power across a range of censoring scenarios. Section 5 illustrates the method using two real datasets. Concluding remarks appear in Section 6.

## 2 Paired Univariate Martingale Residuals

Let (*S_i_*, *T_i_*), *i* = 1, …, *n*, denote i.i.d. paired failure times, and let (*C_i_*, *D_i_*) denote independent, non-informative right-censoring times. We observe

$$X_{i}=min\left(S_{i},C_{i}\right),\;\delta_{S_{i}}=I\left(S_{i}\leq C_{i}\right),$$

$$Y_{i}=min\left(T_{i},D_{i}\right),\;\delta_{T_{i}}=I\left(T_{i}\leq D_{i}\right).$$

To construct rank-based measures of association under censoring, we require transformations of the marginal data that (i) preserve the ordering information contained in the observed survival times, (ii) incorporate censoring through the Kaplan–Meier estimators, and (iii) reduce to a monotone transformation of the original data when no censoring is present. Univariate martingale residuals (UMRs), introduced by Therneau et al.[6], satisfy these requirements and provide a natural basis for extending Spearman’s rank correlation to censored data.

Let ${\overset{ˆ}{H}}_{S}$ and ${\overset{ˆ}{H}}_{T}$ denote the Kaplan–Meier cumulative hazard estimators for the *S* and *T* margins, respectively. The UMRs for the *i*th observation are defined as

(2.1) $$\left(m_{S_{i}},m_{T_{i}}\right)=\left(\delta_{S_{i}}-{\overset{ˆ}{H}}_{S}\left(X_{i}\right),\;\delta_{T_{i}}-{\overset{ˆ}{H}}_{T}\left(Y_{i}\right)\right),$$

for *i* = 1, …, *n*.

### Martingale structure.

The martingale residuals are defined with respect to the counting-process filtration

$${ℱ}_{t}=\sigma \left\{N_{S}(u),Y_{S}(u),N_{T}(u),Y_{T}(u):0\leq u\leq t\right\},$$

where *N_S_* and *Y_S_* denote the event and at-risk processes for the *S* margin (and similarly for *T*). This clarifies the martingale property underlying the UMR construction.

### Monotonicity and rank recovery.

Under Assumptions (A1)–(A4), the Kaplan–Meier cumulative hazard estimators satisfy

$${\overset{ˆ}{H}}_{S}\left(X_{i}\right)=H_{S}\left(S_{i}\right)+o_{\text{a.s.}}(1),\;{\overset{ˆ}{H}}_{T}\left(Y_{i}\right)=H_{T}\left(T_{i}\right)+o_{\text{a.s.}}(1),$$

because $X_{i}=S_{i}$ whenever $\delta_{S_{i}}=1$ and the KM estimator is uniformly strongly consistent. For uncensored observations,

$$m_{S_{i}}=1-H_{S}\left(S_{i}\right)+o_{\text{a.s.}}(1),$$

and the map $x\mapsto 1-H_{S}(x)$ is strictly decreasing on the support of *F_S_*. For censored observations,

$$m_{S_{i}}=-H_{S}\left(C_{i}\right)+o_{\text{a.s.}}(1),$$

and because $C_{i}$ is independent of $S_{i}$ with a continuous distribution, the probability of ties among $\left\{m_{S_{i}}\right\}$ is o(1). Thus the ordering of $\left\{m_{S_{i}}\right\}$ coincides with the ordering of $\left\{S_{i}\right\}$ with probability 1−o(1), and similarly for $\left\{m_{T_{i}}\right\}$. Consequently,

$$\frac{a_{i}}{n}\overset{\text{a.s.}}{\to }F_{S}\left(S_{i}\right),\;\frac{b_{i}}{n}\overset{\text{a.s.}}{\to }F_{T}\left(T_{i}\right),$$

establishing the rank-recovery property used in Theorem 1.

### No-censoring case.

When no censoring is present, $\delta_{S_{i}}=\delta_{T_{i}}=1$ for all *i*, and

$$m_{S_{i}}=1-{\overset{ˆ}{H}}_{S}\left(S_{i}\right),\;m_{T_{i}}=1-{\overset{ˆ}{H}}_{T}\left(T_{i}\right),$$

which are strictly monotone transformations of (*S_i_*, *T_i_*). Thus the UMR ranks coincide with the usual ranks (up to deterministic reversal), and the resulting statistic reduces exactly to the classical Spearman permutation test.

### Technical note.

A full derivation of the Kaplan–Meier survival and cumulative hazard estimators used in (2.1) is provided in the Appendix.

## 3 Inference about the Spearman Rank Correlation Coefficient around UMR’s Under Right Censoring

Let $a_{i}=Rank\left(m_{S_{i}}\right)$ and $b_{i}=Rank\left(m_{T_{i}}\right)$ denote the paired ranks of the UMRs $\left(m_{S_{i}},m_{T_{i}}\right)$ defined in (2.1). The UMR-adjusted Spearman rank correlation coefficient is

(3.1) $$r_{s}\left(m_{S},m_{T}\right)=1-\frac{6\sum_{i=1}^{n}{\left(a_{i}-b_{i}\right)}^{2}}{n\left(n^{2}-1\right)}.$$

### Interpretation and inferential target.

Because the UMRs incorporate censoring through the Kaplan–Meier cumulative hazard estimators, the statistic *r_s_* (*m_S_, m_T_*) should not be interpreted as an estimator of the latent Spearman correlation *ρ_s_* when censoring is present. Rather, it is a *Spearman-like measure of association* computed from the ranked martingale residuals, and serves as a test statistic whose null distribution is consistently estimated by permutation. When no censoring is present, the UMRs reduce to strictly monotone transformations of (*S_i_*, *T_i_*), and therefore *r_s_*(*m_S_, m_T_*) = *r_s_*(*S, T*), recovering the classical Spearman coefficient. Under censoring, the proposed procedure should be viewed as a rank-based test for association between the martingale-residual transforms, preserving the Spearman interpretation in the uncensored limit.

To test *H*_0_ : *ρ_s_* = 0 versus *H*_1_ : *ρ_s_* > 0, we apply the studentized permutation framework[5] to the paired UMRs. The variance estimator is

$${\overset{ˆ}{\tau }}_{n}^{2}=\frac{{\overset{ˆ}{\mu }}_{22}}{{\overset{ˆ}{\mu }}_{20}{\overset{ˆ}{\mu }}_{02}},\;{\overset{ˆ}{\mu }}_{pq}=\frac{1}{n}\sum_{i=1}^{n}{\left(a_{i}-\overset{‾}{a}\right)}^{p}{\left(b_{i}-\overset{‾}{b}\right)}^{q},$$

and the studentized statistic is

$$R_{s}=\frac{r_{s}}{{\overset{ˆ}{\tau }}_{n}}.$$

A permutation *p*-value is obtained by permuting $\left\{b_{1},\ldots ,b_{n}\right\}$, recomputing $R_{s}^{\pi }$ for each permutation, and comparing *R_s_* to its permutation distribution.

#### Summary of the UMR-Based Studentized Permutation Test.

1. Compute the Kaplan–Meier cumulative hazard estimators ${\overset{ˆ}{H}}_{S}$ and ${\overset{ˆ}{H}}_{T}$.
2. Form the univariate martingale residuals
$$m_{S_{i}}=\delta_{S_{i}}-{\overset{ˆ}{H}}_{S}\left(X_{i}\right),\;m_{T_{i}}=\delta_{T_{i}}-{\overset{ˆ}{H}}_{T}\left(Y_{i}\right).$$
3. Rank the residuals to obtain (*a_i_, b_i_*).
4. Compute the studentized statistic $R_{s}=r_{s}/{\overset{ˆ}{\tau }}_{n}$.
5. Permute $\left\{b_{i}\right\}$, recompute $R_{s}^{\pi }$ for each permutation, and compute the permutation *p*-value.

#### Assumptions

We impose the following regularity conditions, which are standard in survival analysis and permutation asymptotics.

(A1) (*S_i_, T_i_*) are i.i.d. latent failure times with continuous marginal distribution functions *F_S_* and *F_T_*.
(A2) The censoring variables (*C_i_, D_i_*) are i.i.d. and independent of (*S_i_, T_i_*), with continuous distributions.
(A3) Under *H*_0_, *S_i_* and *T_i_* are independent.
(A4) The Kaplan–Meier estimators ${\overset{ˆ}{H}}_{S}$ and ${\overset{ˆ}{H}}_{T}$ satisfy uniform strong consistency:
$$\underset{x}{sup}\left|{\overset{ˆ}{H}}_{S}(x)-H_{S}(x)\right|\overset{\text{a.s.}}{\to }0,\;\underset{y}{sup}\left|{\overset{ˆ}{H}}_{T}(y)-H_{T}(y)\right|\overset{\text{a.s.}}{\to }0.$$
(A5) The UMR-based Spearman statistic admits a finite-variance Hoeffding decomposition with a degenerate first-order term under *H*_0_.

##### Remark on Assumption (A5).

Because Theorem 1 establishes that (*a_i_/n, b_i_/n*) converge almost surely to independent (*U, V*) with continuous distributions, the UMR-based statistic inherits the same degeneracy property as the classical Spearman statistic. Formally,

$$E\left\{\psi \left(Z_{i}\right)|S_{i}\right\}=0,\;E\left\{\psi \left(Z_{i}\right)|T_{i}\right\}=0,$$

under *H*_0_, which implies that the linear term in the Hoeffding decomposition vanishes. This places the statistic within the framework of DiCiccio and Romano [7], ensuring the validity of the studentized permutation distribution.

#### Theorem 1 (Asymptotic exchangeability)

**Theorem 1.** *Under Assumptions (A1)–(A4) and H*_0_ : *ρ_s_* = 0, *the normalized ranks of the UMRs satisfy*

$$\frac{a_{i}}{n}\overset{\text{a.s.}}{\to }F_{S}\left(S_{i}\right),\;\frac{b_{i}}{n}\overset{\text{a.s.}}{\to }F_{T}\left(T_{i}\right),$$

and the limits are independent. Consequently, the joint distribution of the ranked pairs $\left\{\left(a_{i},b_{i}\right)\right\}$ is asymptotically invariant under permutations of $\left\{b_{i}\right\}$.

**Proof.** By definition,

$$m_{S_{i}}=\delta_{S_{i}}-{\overset{ˆ}{H}}_{S}\left(X_{i}\right),\;m_{T_{i}}=\delta_{T_{i}}-{\overset{ˆ}{H}}_{T}\left(Y_{i}\right).$$

Assumption (A4) implies

$$m_{S_{i}}=\delta_{S_{i}}-H_{S}\left(S_{i}\right)+o_{\text{a.s.}}(1),\;m_{T_{i}}=\delta_{T_{i}}-H_{T}\left(T_{i}\right)+o_{\text{a.s.}}(1).$$

For uncensored observations, the map $x\mapsto 1-H_{S}(x)$ is strictly monotone on the support of *F_S_*, and similarly for *T*. For censored observations, ties among UMRs occur with probability *o*(1) under continuous margins. Thus the ranks satisfy

$$\frac{a_{i}}{n}\overset{\text{a.s.}}{\to }F_{S}\left(S_{i}\right),\;\frac{b_{i}}{n}\overset{\text{a.s.}}{\to }F_{T}\left(T_{i}\right).$$

Assumption (A3) ensures independence of the limits, establishing asymptotic exchangeability.

#### Theorem 2 (Validity of the permutation distribution)

**Theorem 2.** *Let R_s_ denote the studentized UMR-based Spearman statistic and let* $R_{s}^{\pi }$ *denote the statistic computed after permuting* $\left\{b_{i}\right\}$. *Under Assumptions (A1)–(A5) and H_0_,*

$$\underset{x\in \mathbb{R}}{sup}\left|P\left(R_{s}\leq x\right)-P\left(R_{s}^{\pi }\leq x\right)\right|\to 0.$$

**Proof.** By Assumption (A5), *r_s_* admits a Hoeffding decomposition

$$r_{s}=\frac{1}{n}\sum_{i=1}^{n}\psi \left(Z_{i}\right)+\frac{1}{n(n-1)}\sum_{i\neq j}\phi \left(Z_{i},Z_{j}\right),$$

with a degenerate first-order term under *H_0_*. Studentization removes nuisance scale parameters, yielding

$$R_{s}\overset{d}{\to }N(0,1).$$

By Theorem 1, the ranked pairs are asymptotically exchangeable, so the permutation distribution of the degenerate U-statistic kernel converges to the same Gaussian limit. The general theory[7] then implies the stated result. □

#### Theorem 3 (Asymptotic Type I error control)

**Theorem 3.** *Let* $c_{\alpha }$ *denote the* (1 − *α*) *quantile of the permutation distribution of* $R_{s}^{\pi }$. *Under Assumptions (A1)–(A5) and H*_0_,

$$\underset{n\to \infty }{lim}P\left(R_{s}>c_{\alpha }\right)=\alpha .$$

**Proof.** By Theorem 2,

$$c_{\alpha }\overset{P}{\to }z_{1-\alpha },$$

the (1 − *α*) quantile of the standard normal distribution. Under *H*_0_, Theorem 2 also gives $R_{s}\overset{d}{\to }N(0,1)$. Thus

$$P\left(R_{s}>c_{\alpha }\right)=P\left(R_{s}>z_{1-\alpha }\right)+o(1)=\alpha +o(1),$$

establishing asymptotic Type I error control. □

### Corollary (Reduction to the classical Spearman test).

If no censoring is present, then $\delta_{S_{i}}=\delta_{T_{i}}=1$ for all *i*, and the UMRs reduce to strictly monotone transformations of (*S_i_*, *T_i_*). Their ranks therefore coincide with the usual ranks of the uncensored data, and the statistic *R_s_* and its permutation distribution reduce exactly to the classical studentized permutation test for Spearman’s *ρ_s_*.

## 4 Simulation Study

In this section, we evaluate the operating characteristics of the proposed studentized permutation test for

$$H_{0}:\rho_{s}=0\;\text{versus}\;H_{1}:\rho_{s}>0,$$

using the UMR-based Spearman statistic described in Section 3. All results are based on *B* = 2000 permutations unless otherwise noted.

To our knowledge, no competing permutation-based test for Spearman’s correlation under censoring currently exists. For comparison, we include the modified Kendall concordance statistic of Oakes[3]. Although Oakes’ test is not designed to estimate or test Spearman’s *ρ_s_*, it is one of the few available procedures providing a valid independence test under right censoring. We therefore use it as a benchmark for Type I error and power, while recognizing that its inferential target differs from that of Spearman-type measures. This distinction explains the conservativeness observed in our simulations.

We consider two simulation scenarios based on correlated shifted exponential variables. In both scenarios, the underlying uncensored variables are generated as follows. First, draw $X^{\prime }∼exp(1)$ and $Y^{\prime }∼exp(1)$ independently. Then define

$$X=\left(X^{\prime }-1\right)+2,\;Y=\left(X^{\prime }-1\right)\rho +\left(Y^{\prime }-1\right)\sqrt{1-\rho^{2}}+2,$$

for *ρ* = 0, 0.2, 0.4, 0.6, 0.8. Shifting both variables by 2 ensures positivity and simplifies the censoring mechanism. Sample sizes of *n* = 30, 40, 50, 60 were examined for power comparisons. Sample sizes of *n* = 20, 30, 40, 50, 500, 1000 were examined for Type I error comparisons.

### Scenario 1: Moderate Censoring

Censoring variables *C* and *D* were generated independently from $C^{\prime }∼exp(1)$ and $D^{\prime }∼exp(1)$ and transformed via

$$C=C^{\prime }+2,\;D=D^{\prime }+2,$$

yielding censoring probabilities P(X>C)=P(Y>D)≈0.50.

**Figure 1:**
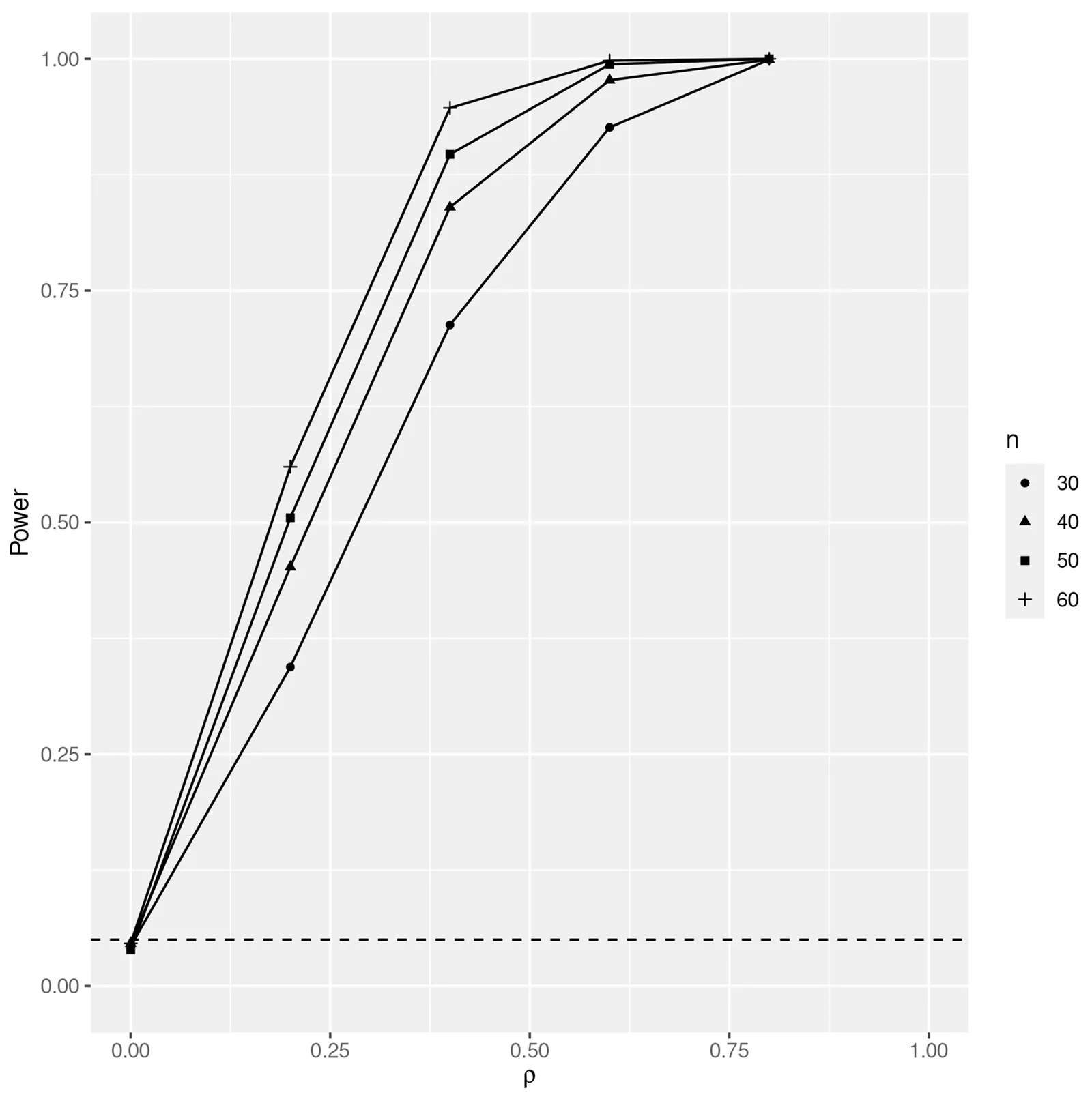
Power curves for Scenario 1 for UMR-based method in Section 4. The horizontal dashed line is the desired Type I error rate at *α* = 0.05.

Figure 1 displays the resulting power curves for the UMR-based test. Type I error is well controlled across all sample sizes, and power increases monotonically with *ρ*, as expected. Table 1 reports estimated Type I error rates for *α* = 0.01, 0.05, 0.10.

For comparison with the classical uncensored setting, we evaluated the power of the Pearson correlation test applied to the fully observed (*X*, *Y*) pairs at *n* = 30. At *ρ* = 0.2, power was 0.344 for the UMR-based test and 0.272 for Pearson; at *ρ* = 0.4, 0.713 versus 0.701; and at *ρ* = 0.6, 0.926 versus 0.972. These results demonstrate that the UMR-based test performs competitively with the classical test even when censoring is present.

**Figure 2:**
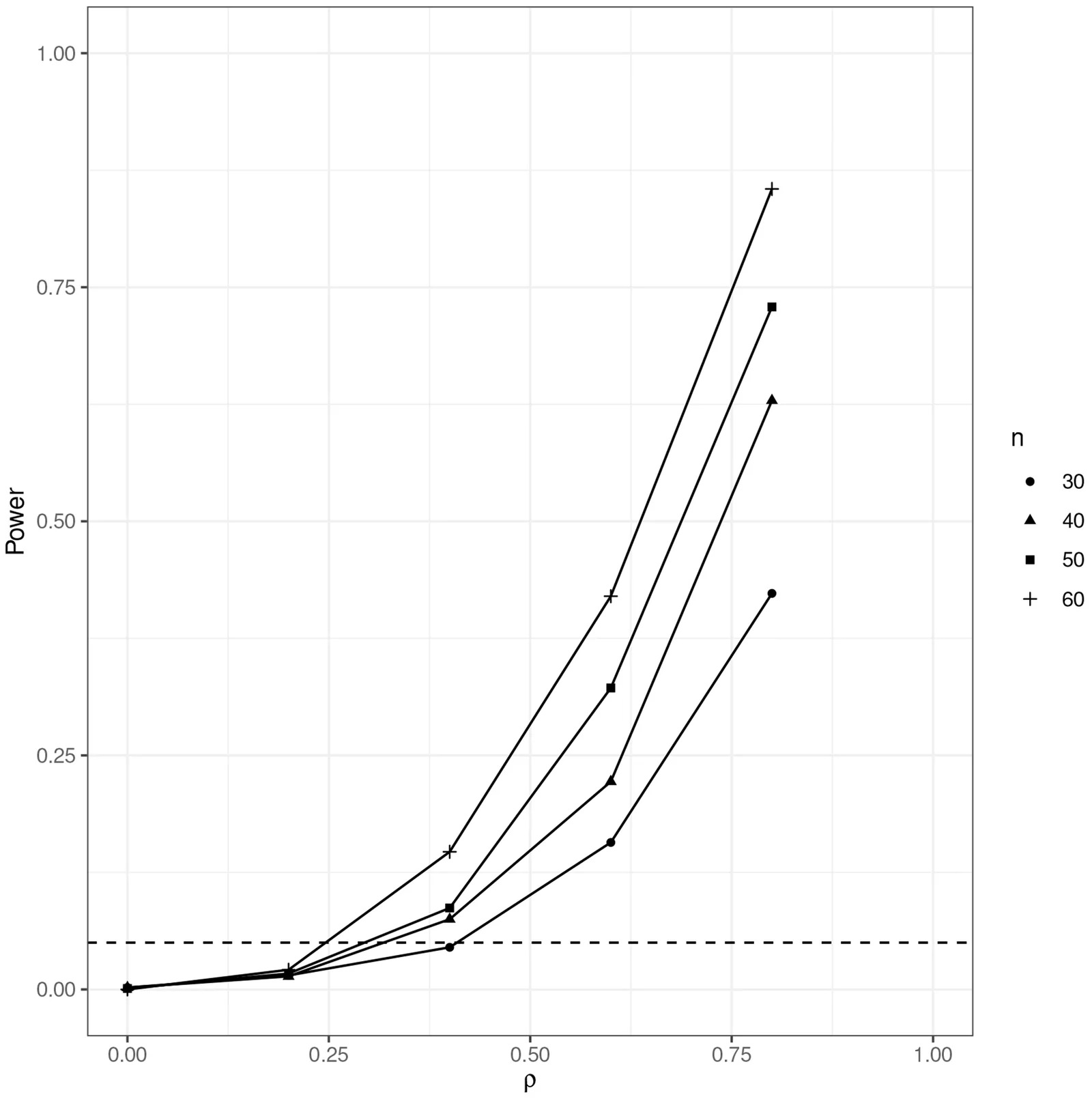
Power curves for Scenario 1 for Kendall’s tau in Section 4. The horizontal dashed line is the desired Type I error rate at *α* = 0.05.

Figure 2 shows that the modified Kendall statistic[3] is markedly conservative, with estimated Type I error below 0.002 across all sample sizes, and substantially lower power than the UMR-based test.

### Scenario 2: Increased Censoring

To examine performance under heavier censoring, we again generated *X* and *Y* as above but modified the censoring mechanism. Censoring variables were drawn from the same exponential distributions but transformed via

$$C=C^{\prime }/2+2,\;D=D^{\prime }/2+2,$$

yielding censoring probabilities of approximately 0.67 for both margins.

**Figure 3:**
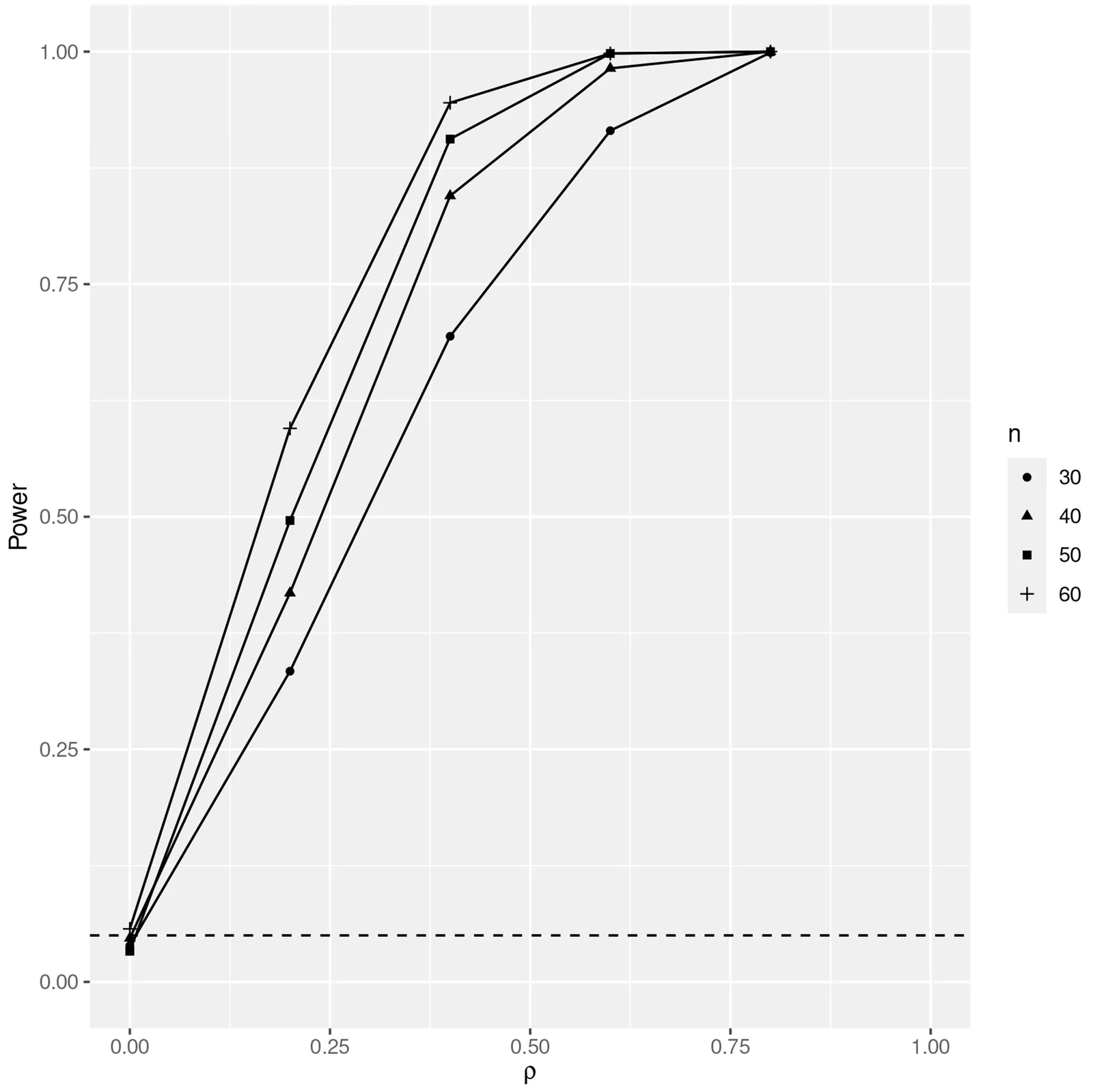
Power curves for Scenario 2 for UMR-based method in Section 4. The horizontal dashed line is the desired Type I error rate at *α* = 0.05.

Figure 3 shows that Type I error remains well controlled and power curves retain the expected monotone behavior. Table 2 summarizes Type I error estimates for the usual significance levels.

At *n* = 30 and *α* = 0.05, the UMR-based test again performs comparably to the Pearson test applied to uncensored data: at *ρ* = 0.2, power was 0.334 (UMR) versus 0.272 (Pearson); at *ρ* = 0.4, 0.694 versus 0.701; at *ρ* = 0.6, 0.915 versus 0.972; and at *ρ* = 0.8, both tests exceeded 0.999.

**Figure 4:**
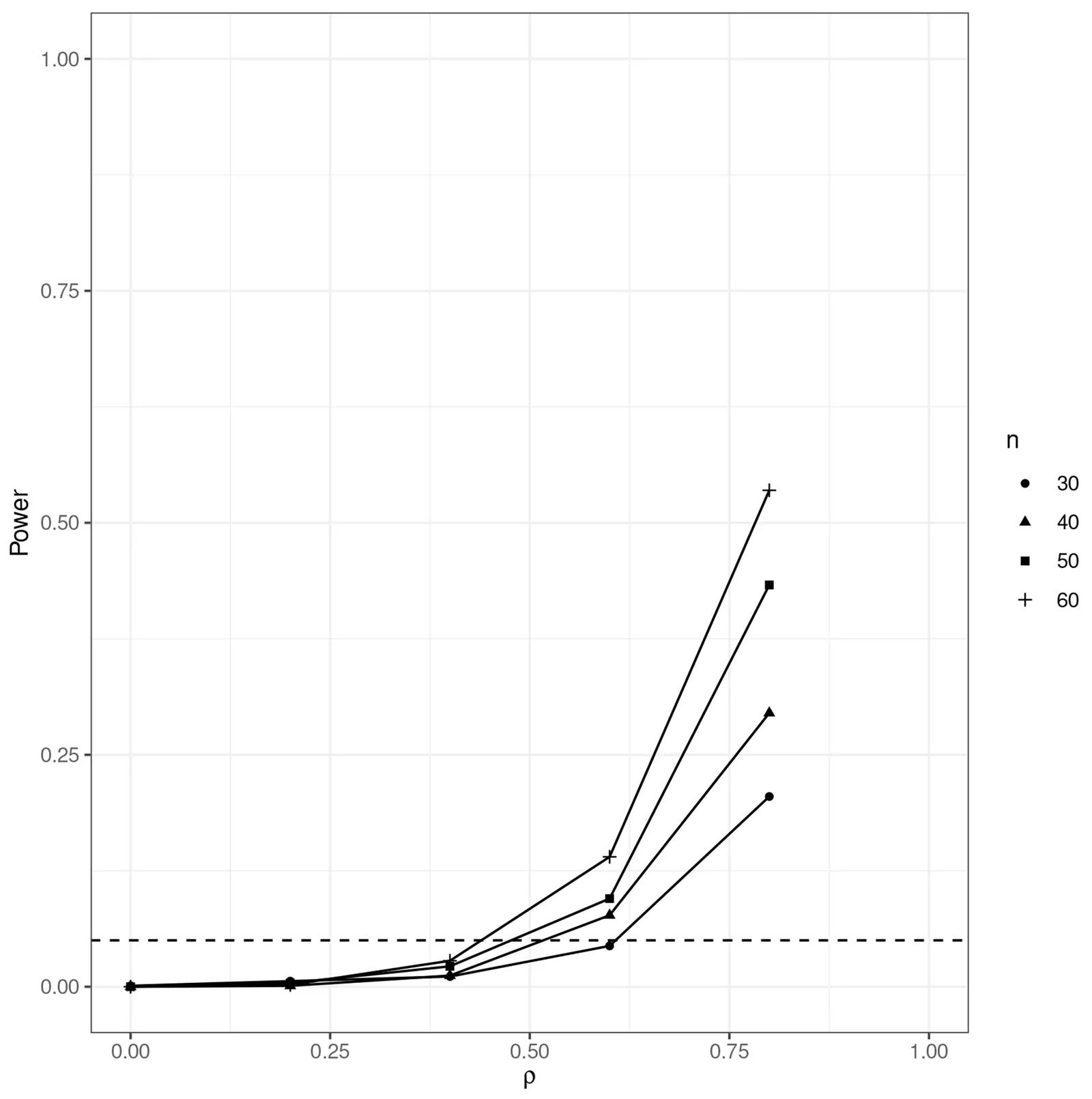
Power curves for Scenario 2 for Kendall’s tau in Section 4. The horizontal dashed line is the desired Type I error rate at *α* = 0.05.

As in Scenario 1, the modified Kendall statistic[3] (Figure 4) remains highly conservative, with Type I error below 0.002 and substantially reduced power relative to the UMR-based test.

### Summary of Findings

Across both censoring scenarios and all sample sizes considered, the UMR-based studentized permutation test exhibits:

- strong Type I error control,
- competitive power relative to the classical Pearson test when censoring is absent,
- substantially higher power than the modified Kendall statistic[3],
- robustness across a range of censoring levels and correlation strengths.

When no censoring is present, the test reduces exactly to the classical studentized permutation test for Spearman’s *ρ_s_*, as shown in the corollary of Section 3. These results demonstrate that the proposed method provides a reliable and powerful tool for testing rank-based association in the presence of right censoring.

## 5 Real Data Examples

In this section, we reanalyze two sets of real bivariate censored data from Akritas and Van Keilegom [8] and Wang and Wells [9] to test *H*_0_ : *ρ_s_* = 0 versus *H*_1_ : *ρ_s_* > 0 at a significance level of *α* = 0.05. Details on the source of the original data are provided in the references.

### 5.1 Survival Days of Skin Grafts in Burn Patients[9]

This dataset includes *n* = 11 paired survival times (in days) for poorly matched skin grafts in burn patients, along with failure indicator variables. The observed survival times are:

s=(37,19,57,93,16,22,20,18,63,29,60),

t=(29,13,15,26,11,17,26,21,43,15,40),

with failure indicators

$$\delta^{x}=(1,1,0,1,1,1,1,1,1,1,0),\;\delta^{y}=(1,1,1,1,1,1,1,1,1,1,1).$$

Using the ranked UMRs, the adjusted Spearman rank correlation coefficient is estimated as *r_s_*(*m_S_, m_T_*) = 0.56. The permutation test yields a p-value of 0.044, which is significant at the *α* = 0.05 level. This indicates a moderate positive association between the paired graft survival times.

### 5.2 Recurrence Times to Infection for Kidney Patients Using Portable Dialysis Equipment[8]

This dataset consists of recurrence times to infection (in days) at the catheter insertion site for *n* = 38 kidney patients using portable dialysis equipment. Each patient has two recorded recurrence times, with some data subject to censoring. Among the 38 patients, 23 have fully observed (doubly uncensored) times, 12 have one censored time (singly censored), and three have both times censored (doubly censored).

The observed survival times are:

s=(8,23,22,447,30,24,7,511,53,15,7,141,96,149,536,17,185,292,22,15,152,402,13,39,12,113,132,34,2,130,27,5,152,190,119,54,6,63),

t=(16,13,28,318,12,245,9,30,196,154,333,8,38,70,25,4,177,114,159,108,562,24,66,46,40,201,156,30,25,26,58,43,30,5,8,16,78,8),

with failure indicators:

$$\delta^{x}=(1,1,1,1,1,1,1,1,1,1,1,1,1,0,1,1,1,1,0,1,1,1,1,1,1,0,1,1,1,1,1,0,1,1,1,0,0,1),$$

$$\delta^{y}=(1,0,1,1,1,1,1,1,1,1,1,0,1,0,0,0,1,1,0,0,1,0,1,0,1,1,1,1,1,1,1,1,1,0,1,0,1,0).$$

Using the ranked UMRs, the adjusted Spearman rank correlation coefficient is estimated as *r_s_*(*m_S_, m_T_*) = 0.33. The permutation test yields a p-value of 0.031, which is significant at the *α* = 0.05 level. This suggests a modest but statistically significant positive association between the paired recurrence times.

## 6 Concluding Remarks

In this note, we have presented a straightforward and robust approach for testing whether the Spearman rank correlation between martingale residuals is zero. The method leverages the studentized permutation framework [5] applied to paired univariate martingale residuals (UMRs), which provide a principled rank-generating transformation that incorporates censoring through the Kaplan–Meier estimators. The resulting test achieves asymptotically correct Type I error control, applies when one or both margins are censored, and reduces exactly to the classical Spearman permutation test when no censoring is present.

Our simulation studies demonstrate that the proposed UMR-based test exhibits strong Type I error control and competitive power across a wide range of censoring levels and correlation strengths. In direct comparisons, the modified Kendall’s tau statistic[3] performs markedly worse, showing both substantial underestimation of the Type I error rate and reduced power relative to the UMR-based Spearman test. Two real-data examples further illustrate the practical utility of the method in biomedical applications where censoring is common.

The UMR-based Spearman test also provides a natural foundation for developing exact tests of independence between *X* and *Y* under censoring, an extension we plan to explore in future work. More broadly, the framework suggests potential generalizations to covariate-adjusted rank correlations and multivariate survival settings. The accompanying R software tools facilitate implementation and support the broader adoption of this methodology in applied research.

## Figures and Tables

**Table 1:** Estimated Type I error control at different sample sizes *n* and significance levels *α*.

n	*α*	Type I error
20	0.01	0.010
20	0.05	0.048
20	0.10	0.100
30	0.01	0.005
30	0.05	0.055
30	0.10	0.089
40	0.01	0.010
40	0.05	0.055
40	0.10	0.083
50	0.01	0.007
50	0.05	0.046
50	0.10	0.100
500	0.01	0.007
500	0.05	0.063
500	0.10	0.101
1000	0.01	0.008
1000	0.05	0.041
1000	0.10	0.120

**Table 2:** Estimated Type I error control at different sample sizes *n* and significance levels *α* under increased censoring.

n	*α*	Type I error
20	0.01	0.013
20	0.05	0.053
20	0.10	0.086
30	0.01	0.011
30	0.05	0.043
30	0.10	0.090
40	0.01	0.011
40	0.05	0.050
40	0.10	0.099
50	0.01	0.006
50	0.05	0.050
50	0.10	0.100
500	0.01	0.008
500	0.05	0.042
500	0.10	0.093
1000	0.01	0.014
1000	0.05	0.056
1000	0.10	0.106

## Data Availability

The Roswell Park Comprehensive Cancer Center IRB has determined that this work does not constitute human subjects research. The raw data used to illustrate the proposed statistical methodology are publicly available and fully contained within Sections 5.1 and 5.2 of the manuscript. The raw datasets are also available at: https://www.researchgate.net/publication/408724150_Data_Used_in_A_Permutation_Test_for_a_Modified_Spearman’s_Rank_Correlation_Using_Martingale-Residual_Ranks_Under_Right_Censoring_1_Survival_Days_of_Skin_Grafts_in_Burn_Patients

## Appendix: Derivation of the Kaplan–Meier and Hazard Components

Let $0<x_{\left(1\right)}<\cdots <x_{\left(f\right)}$ and $0<y_{\left(1\right)}<\cdots <y_{\left(g\right)}$ denote the distinct ordered observed failure times for the *S* and *T* margins, respectively. Following the classical product-limit argument, assume the marginal distributions are discrete with probabilities

$$\theta_{i}=P\left(X=x_{\left(i\right)}\right),\;i=1,\ldots ,f,$$

$$\pi_{j}=P\left(Y=y_{\left(j\right)}\right),\;j=1,\ldots ,g.$$

Define the discrete hazard functions

$$h_{x_{i}}=P\left(X=x_{\left(i\right)}|X\geq x_{\left(i\right)}\right),\;h_{y_{j}}=P\left(Y=y_{\left(j\right)}|Y\geq y_{\left(j\right)}\right),$$

so that

$$\theta_{1}=h_{x_{1}},\;\theta_{2}=\left(1-h_{x_{1}}\right)h_{x_{2}},\;\ldots ,$$

and similarly for *π_j_*.

Let $d_{x_{i}}$ and $r_{x_{i}}$ denote the number of events and number at risk at time *x*_(*i*)_, respectively. The log-likelihood for the *S* margin is

$$log\;L_{S}=\sum_{i=1}^{f}\left[d_{x_{i}}\;log\;h_{x_{i}}+\left(r_{x_{i}}-d_{x_{i}}\right)log\left(1-h_{x_{i}}\right)\right],$$

with an analogous expression for log *L_T_*. Maximizing yields the familiar estimators

$${\overset{ˆ}{h}}_{x_{i}}=\frac{d_{x_{i}}}{r_{x_{i}}},\;{\overset{ˆ}{h}}_{y_{j}}=\frac{d_{y_{j}}}{r_{y_{j}}}.$$

The Kaplan–Meier estimators follow as

$${\overset{ˆ}{S}}_{S}(x)=\prod_{x_{\left(i\right)}\leq x}\left(1-{\overset{ˆ}{h}}_{x_{i}}\right),\;{\overset{ˆ}{S}}_{T}(y)=\prod_{y_{\left(j\right)}\leq y}\left(1-{\overset{ˆ}{h}}_{y_{j}}\right),$$

and the corresponding cumulative hazard estimators are

$${\overset{ˆ}{H}}_{S}(x)=-log\;{\overset{ˆ}{S}}_{S}(x),\;{\overset{ˆ}{H}}_{T}(y)=-log\;{\overset{ˆ}{S}}_{T}(y).$$

These cumulative hazards are used in the definition of the univariate martingale residuals in Equation (2.1).
